# Susceptibility of Methicillin-Resistant Staphylococcus aureus to Five Quinazolinone Antibacterials

**DOI:** 10.1128/AAC.01344-19

**Published:** 2019-12-20

**Authors:** Sara Ceballos, Choon Kim, Yuanyuan Qian, Shahriar Mobashery, Mayland Chang, Carmen Torres

**Affiliations:** a Area of Biochemistry and Molecular Biology, University of La Rioja, Logroño, Spain; b Department of Chemistry and Biochemistry, University of Notre Dame, Notre Dame, Indiana, USA

**Keywords:** methicillin-resistant *Staphylococcus aureus*, MRSA, penicillin-binding proteins, quinazolinones, staphylococci

## Abstract

The *in vitro* activities of five quinazolinone antibacterials, compounds Q1 to Q5, were tested against 210 strains of methicillin-resistant Staphylococcus aureus (MRSA). The MIC_50_/MIC_90_ values (in μg/ml) were as follows: Q1, 0.5/2; Q2, 1/4; Q3, 2/4; Q4, 0.06/0.25; and Q5, 0.125/0.5. Several strains with high MIC values (from 8 to >32 μg/ml) for some of these compounds exhibited amino acid changes in the penicillin-binding proteins, which are targeted by these antibacterials.

## Abstract (precis)

The *in vitro* activities of five quinazolinone antibacterials, compounds Q1 to Q5, were tested against 210 strains of methicillin-resistant Staphylococcus aureus (MRSA). The MIC_50_/MIC_90_ values (in μg/ml) were as follows: Q1, 0.5/2; Q2, 1/4; Q3, 2/4; Q4, 0.06/0.25; and Q5, 0.125/0.5.

## INTRODUCTION

Nosocomial and community-acquired methicillin-resistant Staphylococcus aureus (MRSA) infections remain major clinical problems. Patients with MRSA infections have a 64% higher risk of mortality than those infected with nonresistant bacteria (1). According to the Centers for Disease Control and Prevention, MRSA accounts for >80,000 severe infections and kills >11,000 individuals annually in the United States alone (2). Historically, β-lactam antibiotics have been agents of choice in the treatment of S. aureus infections (3). β-Lactams target inhibition of penicillin-binding proteins (PBPs), which are enzymes of cell wall biosynthesis. S. aureus has four native PBPs, but MRSA has acquired an additional PBP, designated PBP 2a, which affords broad resistance to β-lactam antibiotics in the face of their challenge (4–6).

**FIG 1:**
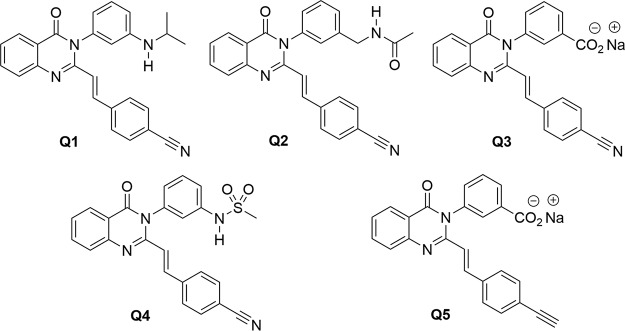
Chemical structures of quinazolinones Q1 to Q5.

We have described the discovery of the quinazolinones as cell wall-active antibacterials with anti-S. aureus activity, including MRSA strains (7). We documented that the quinazolinones target PBP 1 and PBP 2a for inhibition (7). Preliminary structure-activity relationships and descriptions of *in vitro* and *in vivo* activities for the class have been described (7–14). In this report, we investigated the antibacterial activity profile of five quinazolinones of our design, compounds Q1 to Q5 (Fig. 1), against a collection of 210 MRSA strains (108 strains from the United States and 102 from Spain). The collection encompasses distinct clonal complexes and 54 MRSA strains with additional mechanisms of antimicrobial resistance, including resistance to the second-generation penicillin methicillin through the *mecC* gene and resistance to vancomycin or linezolid (Table 1). The 108 strains from the United States were obtained through BEI Resources. The 102 Spanish strains were obtained from four hospitals in three different regions (Aragón, La Rioja, and Madrid) (*n* = 94) and from animal, food, and water origins (*n* = 8, mostly with *mec*C mechanism).

**TABLE 1:** Susceptibility of 210 MRSA strains, including S. aureus NCTC8325, to quinazolinones 1 to 5, ceftaroline, linezolid, and vancomycin

Antibacterial agent	MIC for total MRSA population (*n* = 210) (μg/ml)			MIC range for MRSA with following resistance mechanisms^*a*^ (μg/ml):					MIC for MSSA^*b*^ NCTC8325 (μg/ml)
	Range	MIC_50_	MIC_90_	VRSA (*n* = 15)	VISA (*n* = 20)	hVISA (*n* = 6)	*mecC*-MRSA (*n* = 7)	LRSA (*n* = 6)	
Ceftaroline	0.125 to 2	0.5	1	0.25 to 2	0.25 to 2	0.5 to 1	0.5 to 1	0.5 to 2	0.125
Linezolid	0.5 to >16	2	2	1 to 2	0.5 to 2	0.5 to 2	1 to 2	8 to >16	1
Vancomycin				>32	4 to 8	2 to 8	1 to 2	1 to 4	1
1	0.03 to >32	0.5	2	0.125 to 2	0.25 to 4	0.125 to 4	0.25 to 2	0.25 to 1	0.25
2	0.06 to >32	1	4	0.125 to 8	0.125 to 8	0.25 to 2	0.5 to 2	1 to 4	0.5
3	0.5 to 16	2	4	2 to 16	0.5 to 16	0.5 to 2	1 to 2	1 to 4	1
4	≤0.03 to >32	0.06	0.25	0.06 to 4	0.03 to >32	≤0.03 to 0.125	≤0.03 to 0.06	0.06 to 0.25	0.25
5	≤0.03 to >32	0.125	0.5	0.06 to 2	≤0.03 to 1	≤0.03 to 0.125	0.06 to 0.25	0.06 to 0.25	0.125

a VRSA, vancomycin-resistant S. aureus; VISA, vancomycin-intermediate S. aureus; hVISA, heterogeneous vancomycin-intermediate S. aureus; LRSA, linezolid-resistant S. aureus.

b MSSA, methicillin-susceptible S. aureus. MIC values for ATCC 29213 (quality control strain) were in the range of those shown by CLSI for ceftaroline, linezolid, and vancomycin.

The microdilution method supplemented with 2% NaCl was used to determine the MIC values of quinazolinones Q1 to Q5 (15), using S. aureus NCTC8325 as a reference and a quality-control strain (MIC ranges for Q1 to Q5, 0.125 to 1 μg/ml) (Table 1). Two clinically used anti-MRSA agents, ceftaroline (a β-lactam) and linezolid (an oxazolidinone), were included for the purpose of comparison. Vancomycin (a glycopeptide antibiotic) was included for vancomycin-resistant S. aureus (VRSA), vancomycin-intermediate S. aureus (VISA), and heterogeneous vancomycin-intermediate S. aureus isolates. The MIC ranges for the five quinazolinones with the 210 MRSA strains and the values for MIC_50_ and MIC_90_ are shown in Table 1. The range of quinazolinone MIC values for the 210 MRSA strains was broad, from ≤0.03 to >32 μg/ml. High MICs for the quinazolinones of >32, 16, and 8 μg/ml were observed in 15, 2, and 18 strains, respectively. The MIC_50_/MIC_90_ values (in μg/ml) of the quinazolinones were as follows: Q1, 0.5/2; Q2, 1/4; Q3, 2/4; Q4, 0.06/0.25; and Q5, 0.125/0.5. Notwithstanding the broad MIC ranges for Q4 and Q5, note that compound Q4 MIC_50_/MIC_90_ values were 8- to 4-fold and 32- to 8-fold lower than those of ceftaroline and linezolid, respectively, and compound Q5 MIC_50_/MIC_90_ values were 4- to 2-fold and 16- to 4-fold lower than those of ceftaroline and linezolid, respectively (Table 1). For *mecC*-dependent MRSA and linezolid-resistant S. aureus (LRSA) strains, all quinazolinones had MIC values of ≤2 μg/ml (except for two LRSA strains with MICs of 4 μg/ml). Quinazolinones Q4 and Q5 showed excellent MICs of ≤1 μg/ml against the collected strains with few exceptions: for Q4, one VISA isolate with >32 μg/ml and one VRSA isolate with 4 μg/ml; and for Q5, two MRSA isolates with >32 μg/ml and one VRSA isolate with 2 μg/ml.

Four MRSA strains (C1657, C5287, K399, and C2878) that showed high MICs (>32 μg/ml) for the quinazolinones were selected for sequence analysis of *pbp1*, *pbp2*, *mecA*, *pbp3*, and *pbp4* genes by PCR and DNA sequencing (16, 17); these are the five PBP genes in MRSA. The corresponding amino acid sequences were compared with reference sequences available in the NCBI database: MRSA strain N315 for the *mecA* gene (GenBank accession number BA000018.3) and methicillin-susceptible S. aureus (MSSA) strain NCTC 8325 for the *pbp1*, *pbp2*, *pbp*3, and *pbp4* genes (GenBank accession number LS483365.1). The resultant variations in the amino acids of the proteins are listed in Table 2, in addition to the MIC values for the β-lactams ceftaroline, ceftobiprole, meropenem, and piperacillin-tazobactam in these MRSA strains. The clonal complexes (CC) of these four selected strains were known to belong to CC1, CC8, CC247, and CC398, respectively (18–20). Strain C1657, with an MIC for compound Q2 of >32 μg/ml, showed amino acid substitutions in all PBPs except PBP 2. Strain C5287 (MIC for Q1, >32 μg/ml) exhibited a single amino acid substitution in PBP 2. Strain K399 (MICs for Q1, Q2, and Q5, >32 μg/ml) showed one substitution in PBP 2a (G246E) and none in the other PBPs. Finally, strain C2878 (MICs for Q1, Q2, and Q5, >32 μg/ml) exhibited amino acid changes in all PBPs except PBP 3. The relationship between the amino acid substitutions and the quinazolinone reduced activity is not clear and should be further investigated. However, mutations in PBP 1 and PBP 2a are of special interest, in that these two PBPs are targets of quinazolinones (7, 14).

**TABLE 2:** Amino acid changes of PBPs in MRSA strains that showed high MIC values (>32 μg/ml) to quinazolinones

Strain	*spa*	MLST	CC	MIC for β-lactams^*a*^ (μg/ml)	Compound with MIC >32 μg/ml	Location of amino acid substitutions for:				
						PBP 1	PBP 2	PBP 2a	PBP 3	PBP 4
C1657	t127	ST1	CC1	CPT, 0.5; BPR, 1; TZP, >256; MEM, >32	Q2	V617M	None	G246E	H556L	T189S
C5287	t008	ST8	CC8	CPT, 0.5; BPR, 1; TZP, >256; MEM, >32	Q1	None	R30K	None	None	None
K399	t051	ST247	CC247	CPT, 1; BPR, 2; TZP, >256; MEM, >32	Q1, Q2, Q5	None	None	G246E	None	None
C2878	t1451	ST398	CC398	CPT, 1; BPR, 1; TZP, >256; MEM, 6	Q1, Q2, Q5	F465L, D480E, D662N, S664T	D270E, T439V, D489E, T691A	S225R	None	E398A

a TZP, piperacillin-tazobactam; MEM, meropenem; CPT, ceftaroline; BPR, ceftobiprole.

Quinazolinone Q5 has shown similar *in vivo* activity to the closely related Q3 in mouse models of infection by MRSA (9). Whereas the MIC for MRSA NRS70, the strain used in mouse neutropenic thigh infection, is 8-fold lower for Q5 than for Q3, the plasma protein binding of Q5 is very high (99.6% ± 0.04% for Q5 versus 96.5% ± 0.7% for Q3) (9), decreasing the *in vivo* efficacy of Q5. Q4 has slightly lower MICs (Table 1); however, its PK properties are poor, resulting in low systemic exposure and very high clearance in mice (9). As a result, Q4 shows poor efficacy in the mouse peritonitis MRSA model of infection (9). Thus, both compounds Q3 and Q5 were selected for susceptibility testing (microdilution method) with 32 additional methicillin-susceptible and methicillin-resistant non-*aureus* staphylococci isolates, including 4 linezolid-resistant Staphylococcus epidermidis and Staphylococcus haemolyticus (Table 3). Linezolid MIC was tested by the microdilution method in this study. All staphylococci included in the study belonged to the strain collection of the University of La Rioja (Spain). The range of MICs for Q5 in all Staphylococcus pseudintermedius strains was 4 to 16 μg/ml, with only one methicillin-resistant strain showing a value of 16 μg/ml. Within the coagulase-negative staphylococci (CoNS), all S. epidermidis and S. haemolyticus strains showed MIC values for Q5 of ≤0.125 μg/ml, and the Staphylococcus saprophyticus strain exhibited an MIC of 2 μg/ml. The higher MIC of S. saprophyticus for the quinazolinones may be explained by the fact that methicillin-sensitive S. saprophyticus is resistant to most of the antibiotics used for treatment of urinary tract infections, including ceftriaxone (21). Accordingly, compound Q5 appears to be highly effective against some CoNS, such as S. epidermidis and S. haemolyticus, including linezolid-resistant ones, which are important clinically as opportunistic pathogens causing catheter-associated urinary tract or bloodstream infections (22–24). The activity of Q3 in non-*aureus* staphylococci was lower (Table 3).

**TABLE 3:** MIC for quinazolinones Q3, Q5, and linezolid against non-*aureus* staphylococci

Species^*a*^	No. of strains tested	MIC range (μg/ml) for:		
		Q3	Q5	Linezolid
MR S. pseudintermedius	9	16 to 32	4 to 16	0.5 to 1
MS S. pseudintermedius	2	8	4 to 8	0.5
MR S. epidermidis	10	0.06 to >32	≤0.03 to 0.06	0.25 to 32^*b*^
MR S. haemolyticus	10	0.125 to >32	≤0.03 to 0.125	0.5 to 16^*b*^
MS S. saprophyticus	1	>32	2	1

a MR, methicillin resistant; MS, methicillin susceptible.

b Two MR S. epidermidis and two MR S. haemolyticus were linezolid resistant.

In summary, the quinazolinones have excellent *in vitro* activity against a broad range of MRSA strains. Quinazolinone Q5 stands out for its low MIC_50_/MIC_90_ values against S. aureus isolates and its high antibacterial activity against other staphylococcal species.
